# The Gems and Pitfalls of Inflammatory Breast Disease: An Illustrated Review of Mastitis. What the Radiologist Should Know

**DOI:** 10.1155/rrp/7490766

**Published:** 2026-07-31

**Authors:** Cristiana Boldrini, Micol Bottalico, Francesca L. Lia, Paolo Belli

**Affiliations:** ^1^ Department of Diagnostic Imaging, Oncological Radiotherapy, and Hematology, Diagnostic Imaging Area, Fondazione Policlinico Universitario Agostino Gemelli IRCCS, Rome, Italy

**Keywords:** diabetic mastopathy, idiopathic granulomatous mastitis, infectious mastitis, inflammatory breast cancer, lactational mastitis, mastitis, mondor’s disease, non-infectious mastitis, non-lactational infectious mastitis, periductal mastitis

## Abstract

Mastitis must be considered as a heterogeneous group of inflammatory breast conditions with varying aetiologies, clinical presentations, and management. The primary objective of this pictorial review is to clarify the role of imaging in diagnosis of mastitis, and, above all, in the differential diagnosis of breast cancer (BC), particularly inflammatory cancer. The main literature regarding mastitis published in the PubMed database over the past 18 years was examined. Mastitis is classified into infectious and noninfectious forms, including inflammatory conditions associated with or overlapping with cancer. Infectious mastitis, more common in women of childbearing age and during breastfeeding, is generally caused by bacteria (especially *Staphylococcus aureus*). Ultrasound is the first‐line examination to confirm the inflammatory process and identify any abscess clusters, while mammography has a limited role in the acute phase; MRI may be useful in complex or atypical cases. Noninfectious mastitis includes forms such as periductal mastitis, idiopathic granulomatous mastitis, and inflammatory reactions to foreign bodies or trauma. They can clinically and radiologically mimic cancer. Imaging often shows skin thickening, focal or diffuse parenchymal changes, and irregular hypoechoic areas, sometimes requiring biopsy for a definitive diagnosis. Inflammatory BC is a rare but aggressive entity that can present with clinical signs similar to mastitis. Failure to respond to antibiotic therapy, persistent clinical signs, and certain imaging patterns (especially MRI) should always raise suspicion of cancer and prompt further histological investigations. In conclusion, in the context of inflammatory breast pathology, the radiologist plays a key role in recognizing signs suggestive of benign or malignant disease, ensuring the appropriate management.

**Highlights**
1. Mastitis is classified into infectious and noninfectious forms, including inflammatory breast cancer.
2. Infectious mastitis, more common during childbearing age, is generally caused by bacteria (especially *Staphylococcus aureus*).
3. Noninfectious mastitis can clinically and radiologically mimic cancer.
4. Inflammatory breast cancer is a rare and should be suspected in case of failure of antibiotic therapy and certain imaging.

## 1. Introduction

Mastitis refers to an inflammatory condition affecting the breast, with a range of underlying causes. According to Kamal’s classification, breast inflammation can be grouped into three main categories: infectious mastitis, noninfectious mastitis, and inflammation associated with breast malignancy. [1]

Infectious mastitis includes both breast‐specific and nonspecific infections, which may arise as primary conditions or as complications of pre‐existing breast disorders. This type of mastitis is most frequently observed during the reproductive years, particularly in association with lactation.

Noninfectious mastitis comprises a group of inflammatory conditions of the breast that are not caused by microbial pathogens. These may result from chemical or aseptic processes and typically do not present with the acute symptoms seen in infectious cases of mastitis. As such, they often do not respond to antibiotic therapy.

The third category, and the most clinically significant because of its prognostic and socio‐health implications, is malignant mastitis. This form is typically associated with inflammatory breast carcinoma or, more rarely, a malignant breast abscess. Due to its association with cancer, it represents the most severe and prognostically concerning type of breast inflammation [1].

The main literature regarding inflammatory conditions of the breast (reviews and case reports) published in the PubMed database over the past 18 years was examined, using as keywords the following terms: ‘mastitis’, ‘inflammation of the breast’, ‘lactation mastitis’, ‘infectious mastitis’, ‘tuberculosis of the breast’, ‘hydatidosis of the breast’, ‘cysticercosis of the breast’, ‘filariasis of the breast’, ‘periductal mastitis’, ‘idiopathic granulomatous mastitis’, ‘diabetic mastopathy’, ‘Mondor’s disease’, ‘inflammatory breast cancer’, ‘breast abscess’, ‘breast cancer‐abscess’. Many conditions are relatively rare in the general population; therefore, the case report category has also been used as a source of crucial information on the clinical and radiological characteristics of mastitis. The images relating to the various breast changes were collected partly from our hospital archives and refer to female patients seen between 2017 and 2025, partly from published literature and are licensed under the CC‐BY 4.0 licence (https://creativecommons.org/licenses/by/4.0/). Table 1 provides an overview of the pathologies treated in this narrative illustrated review.

**TABLE 1:** The classification of mastitis into three categories proposed by Kamal et al. [1], and presented in this review.

Infectious mastitis	Noninfectious mastitis	Inflammatory breast cancer (IBC)
Lactational mastitis	Periductal mastitis	
Nonlactational infectious mastitis	Idiopathic granulomatous mastitis	
Tuberculous mastitis	Diabetic mastopathy	
Hydatid disease	Mondor’s disease	
Cysticercosis		
Filariasis		

The three categories of mastitis (infectious, noninfectious, and malignant) will be considered separately within three dedicated macro‐paragraphs, and for each category, the clinical and radiological characteristics of the main pathological entities will be listed. Furthermore, iconographic documentation of the individual clinical conditions will be provided with reference to the main breast imaging techniques (mammography, ultrasound, and magnetic resonance imaging [MRI], and, where available, also computed tomography).

## 2. Infectious Mastitis

Infectious mastitis is commonly categorized into two subtypes: lactational (puerperal) mastitis, which occurs in breastfeeding women, and nonlactational mastitis, which arises independently of the lactation period.

### 2.1. Lactational Mastitis

Lactational mastitis is an inflammatory condition of the mammary gland that occurs during the breastfeeding period. Its incidence has been reported in approximately one in four women during the first 25 weeks postpartum [2].

The underlying causes of lactational mastitis are multifactorial, involving both mechanical and microbial factors. Commonly, the condition originates when bacteria enter via damaged nipple epithelium, such as cracks or fissures, allowing pathogens to migrate into the mammary ducts and glandular tissue. The most frequently identified causative pathogen in acute mastitis is *Staphylococcus aureus*.

Blocked milk ducts are also recognized as a significant risk factor for mastitis. They can lead to breast engorgement, milk stasis, and subsequent inflammation. Ductal obstruction may result from various factors, including missed feeds, poor infant latch leading to inadequate milk removal, or mechanical pressure from tight‐fitting clothing. These same factors are independently associated with an increased likelihood of mastitis [3, 4].

Emerging research highlights the role of mammary dysbiosis, an imbalance in the breast microbiome, as a contributor to mastitis. Reduced microbial diversity and over‐representation of aerotolerant organisms, such as *Staphylococcus* (including *S. epidermidis*), have been observed in both acute and subacute mastitis cases. These species often proliferate within ductal biofilms, narrowing ducts and exacerbating milk stasis without necessarily initiating classic infection [5].

Lactational mastitis typically presents with localized breast tenderness, swelling, and erythema of a painful inflamed region of a single breast. These local symptoms are frequently associated with systemic symptoms, including fever, fatigue, and flu‐like symptoms. In certain cases, the condition may initially be subclinical, with nonspecific clinical findings, potentially delaying diagnosis. Additionally, reactive lymphadenopathy may occur, particularly within the ipsilateral axillary region, leading to axillary discomfort or swelling [6].

The diagnosis of lactational mastitis is primarily clinical, based on the patient’s history and physical examination findings. In most cases, no additional investigations are required at initial presentation. However, if there is no clinical improvement within 48 h of appropriate first‐line antibiotic therapy, further evaluation is warranted. In such cases, milk culture may be indicated to identify potential antibiotic‐resistant microorganisms (bacteria), such as methicillin‐resistant *Staphylococcus aureus* (MRSA), particularly in patients with persistent symptoms. Additionally, if a breast abscess is suspected, a breast ultrasound should be performed [7].

**FIGURE 1:**
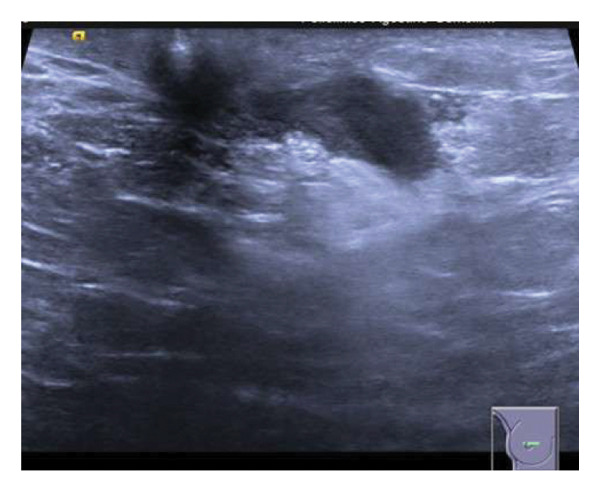
A 36‐year‐old woman presented with right breast pain during breastfeeding. Ultrasound reveals a retro areolar nodular lesion with irregular margins and hypoechoic appearance, initially suggestive of a breast abscess.

**FIGURE 2:**
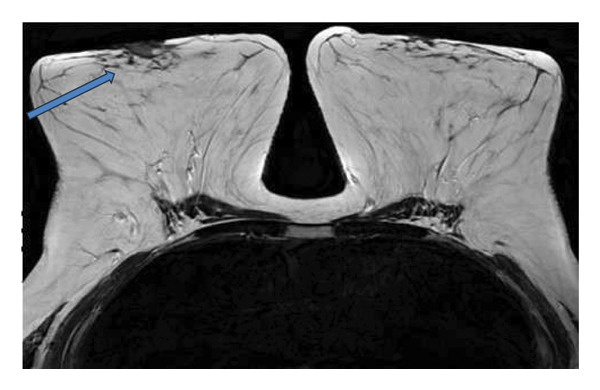
The patient (the same as in Figure 1) underwent breast ce‐MRI. The T2‐weighted sequence shows a right retro areolar nodular lesion containing a small fluid–debris collection (arrow in 2). The post‐contrast T1 sequence demonstrates a mass‐like enhancement in the right retro areolar region extending to the nipple, with a focal area of low signal intensity surrounded by enhancing margins, suggestive for a breast abscess (arrow in 3).

**FIGURE 3:**
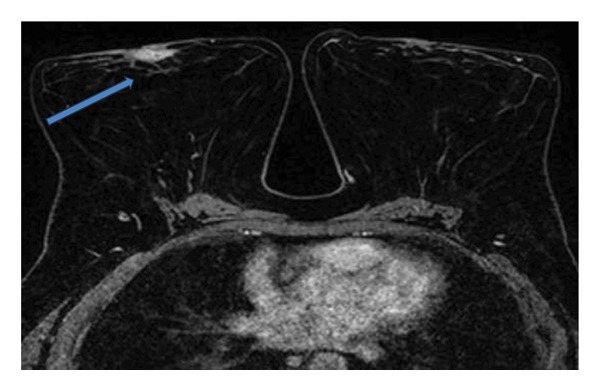
Same patient as in Figure 1‐2. The post‐contrast T1 sequence demonstrates a mass‐like enhancement in the right retro areolar region extending to the nipple, with a focal area of low signal intensity surrounded by enhancing margins, suggestive for a breast abscess (arrow in 3). The post‐contrast T1 sequence demonstrates a mass‐like enhancement in the right retro areolar region extending to the nipple, with a focal area of low signal intensity surrounded by enhancing margins, suggestive for a breast abscess (arrow in 3).

On ultrasound examination, lactational mastitis typically manifests as diffuse skin thickening and echogenic heterogeneous breast parenchyma, reflecting underlying inflammation and oedema. Colour Doppler imaging often reveals increased vascularity within the affected tissue. When abscess formation occurs, it appears as a focal fluid collection characterized by an irregular, hypoechoic mass, with poorly defined margins and heterogeneous internal echoes. These collections commonly demonstrate a posterior acoustic enhancement. The presence of mobile internal debris within the fluid collection is an important distinguishing feature that aids in differentiating a drainable abscess from an inflammatory phlegmon. Tenderness upon probe palpation is frequently observed during the ultrasound examination. Additionally, reactive enlargement of ipsilateral axillary lymph nodes may be present, corresponding with the regional inflammatory response. Most of these abscesses will resolve with antibiotics, ultrasound‐guided needle aspiration, or surgical drainage [8, 9] (Figures 1, 2, and 3).

### 2.2. Nonlactational Infectious Mastitis

Infectious mastitis and breast abscesses are predominantly caused by skin‐colonizing bacteria, with *Staphylococcus aureus* being the most frequently isolated pathogen, followed by *coagulase-negative staphylococci*. Breast infections can be polymicrobial, involving both aerobic and anaerobic bacteria, especially in chronic or recurrent abscess cases. Less common pathogens include mycobacteria (both *Mycobacterium tuberculosis* and other atypical mycobacteria), fungi such as *Candida* and *Cryptococcus* species, and parasites [10].

Clinical diagnosis is typically based on patient history and characteristic symptoms, including localized breast pain, erythema, swelling, nipple discharge, and systemic signs like fever and malaise.

Ultrasound plays a critical role in the evaluation of patients with breast inflammation. It should be performed systematically, with bilateral breast assessment and careful evaluation of the axillary lymph nodes. Important findings for the differential diagnosis are signs of inflammation, including oedema, skin thickening, ductal dilatation, or abscess formation. Furthermore, ultrasound serves as a valuable tool for guiding percutaneous procedures, including fine‐needle aspiration or core needle biopsy (CNB), which are essential for both diagnosis and therapeutic planning. It is also highly effective for guiding lymph node biopsies in patients with suspected nodal involvement.

In women over 30 presenting with signs of breast inflammation, mammography is recommended. While inflammation may sometimes significantly limit the quality of mammograms due to suboptimal compression, the resulting images can still offer key insights for diagnosis and management. Bilateral mammography is important, as comparison with the contralateral unaffected breast enhances the ability to detect subtle abnormalities and can help identify disease not yet clinically apparent [11].

Galactography is a specialized imaging procedure traditionally utilized in the evaluation of patients presenting with pathologic nipple discharge that involves the mammographic visualization of a pathological duct after the instillation of iodinated contrast medium. This technique can reveal intraductal filling defects, ductal irregularities, obstruction, or ductal dilation. Galactography has long been the gold standard for assessing nipple discharge. However, as an invasive test that can cause discomfort and has several limitations, such as difficulty cannulating the duct, the risk of periductal contrast extravasation, and contrast‐related adverse effects, it is increasingly considered obsolete in modern multimodal breast imaging. Consequently, breast MRI has replaced galactography in many centres [12, 13].

Breast MRI with contrast is reserved for specific clinical scenarios, particularly when inflammatory breast carcinoma is suspected or confirmed. Recent advances have highlighted the role of diffusion‐weighted imaging (DWI) MRI in differentiating abscesses from necrotic tumours. Abscesses typically demonstrate marked diffusion restriction with low apparent diffusion coefficient (ADC) values centrally, aiding in noninvasive differential diagnosis [14].

**FIGURE 4:**
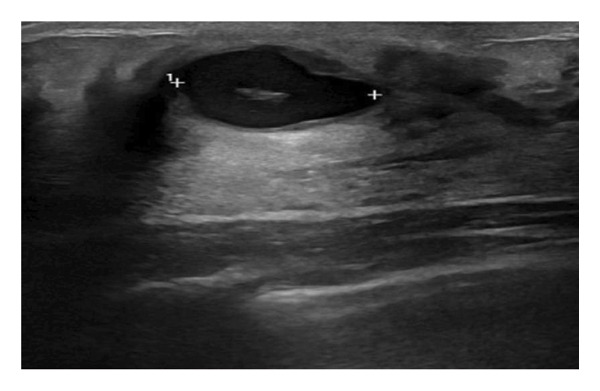
A 12‐year‐old patient presented with right breast pain. The ultrasound shows in the retro areolar area an anechoic nodular formation with posterior wall reinforcement and thickened walls due to abscessation, which resolved after antibiotic therapy.

In routine uncomplicated mastitis, biopsy is generally not indicated. However, in cases of abscess formation, atypical clinical features, uncertain diagnosis, or recurrent infections, tissue sampling may be warranted to exclude malignancy or uncommon infectious aetiologies [10] (Figure 4).

### 2.3. Tuberculous Mastitis (TM)

Tuberculosis of the breast is a relatively rare form of extrapulmonary tuberculosis caused by infection with *Mycobacterium tuberculosis* complex. While the lungs are the primary site of infection for tuberculosis, dissemination to other organs including the breast can occur via lymphatic, haematogenous, or contiguous spread. This condition most commonly affects women of reproductive age, particularly multiparous and lactating females between 20 and 40 years old.

Breast tuberculosis can be classified into two broad categories: primary and secondary. Primary breast tuberculosis is exceptionally rare and arises when the bacilli directly inoculate the breast tissue through minor skin abrasions, fissures in the nipple, or through ductal openings. Secondary breast tuberculosis is more prevalent and often results from retrograde spread of infection from the ipsilateral axillary lymph nodes, which themselves become infected from a primary pulmonary or systemic focus. Haematogenous dissemination, although less common, also represents a possible route of breast involvement [15].

The clinical presentation of breast tuberculosis is highly variable, making diagnosis challenging. Most patients report having a palpable breast mass that is typically painless, firm to hard, irregular in shape, and may be fixed to the overlying skin or underlying chest wall. These lesions most commonly involve the upper outer quadrant or the central portion of the breast. Other clinical features include nipple retraction, skin changes such as erythema, induration, or ulceration, localized swelling, inflammatory signs, and sinus tract or fistula formation in advanced cases. Axillary lymphadenopathy is often present, while nipple discharge is a relatively uncommon symptom [16].

Radiological evaluation plays a critical role in the assessment and differential diagnosis of breast tuberculosis. Imaging findings can be divided into three morphological types: nodular, diffuse, and sclerosing patterns.

The *nodular* form is characterized by one or more well‐circumscribed, slow‐growing lesions that correspond pathologically to noncaseating granulomas. Patients with a strong immune response and less virulent bacilli may develop well‐defined granulomas without caseation, appearing as smoothly marginated nodules on mammography and ultrasound. On sonographic examination, these lesions typically exhibit regular contours, homogenous hypoechogenicity, and posterior acoustic enhancement.

The *diffuse* form of breast tuberculosis is associated with a more aggressive infectious process. It typically manifests as multiple intercommunicating abscesses within the breast parenchyma, causing poorly defined, irregular masses with indistinct margins. This pattern reflects extensive tissue destruction and suppuration, with the abscesses often infiltrating the surrounding fibro‐fatty tissue. Associated inflammatory oedema further obscures lesion borders on imaging, complicating differentiation from malignancies or other inflammatory breast diseases.

The *sclerosing* form predominates when fibrotic reaction outweighs the necrotic process. In this pattern, extensive fibrosis results in asymmetry between the breasts, increased mammographic density, reduction in breast volume, and skin and nipple retraction. Fibrosis involves Cooper’s ligaments and leads to architectural distortion, atrophy, and retraction of the breast glandular tissue, manifesting clinically as skin tethering and contour irregularity [17].

Histopathological confirmation remains the definitive diagnostic modality for breast tuberculosis. Tissue biopsy, obtained through fine needle aspiration cytology (FNAC), CNB, or excisional biopsy, typically reveals epithelioid cell granulomas with central caseous necrosis, surrounded by aggregates of lymphocytes and multinucleated Langhans giant cells. In some cases, granulomatous inflammation without caseation may be observed, especially in early or treated disease. Microbiological analysis, including Ziehl–Neelsen staining and mycobacterial culture, can provide confirmatory evidence of *Mycobacterium tuberculosis*, though these tests may be negative due to the paucibacillary nature of the disease.

The therapeutic approach to breast tuberculosis mirrors that of pulmonary and other extrapulmonary forms of tuberculosis. Standard antitubercular therapy (ATT) consists of a multidrug regimen administered over a period of 6–18 months, depending on disease severity and patient response. Surgical intervention is generally reserved for complicated cases, including those with persistent abscess formation requiring drainage, extensive fibrotic masses causing deformity or pain, or failure to respond adequately to medical therapy [18].

### 2.4. Other Rare Infections

#### 2.4.1. Hydatid Disease

Echinococcal disease is a zoonotic cystic parasitic infection primarily caused by *Echinococcus granulosus*. It is endemic in many regions worldwide and predominantly affects the liver and lungs; involvement of other organs, including the breast, is rare. Hydatid cysts of the breast represent an uncommon clinical entity. Affected patients typically present between 30 and 50 years of age with a painless and progressively enlarging breast mass.

Ultrasound plays a crucial role in evaluating the internal morphology of the cyst, which varies according to the stage and activity of the disease. Sonographic appearances can range from purely cystic lesions to predominantly solid masses as the cyst evolves. Internal echoes correspond to hydatid sand, comprising detached membranes, hooklets, and debris, features characteristic of hydatid disease as seen in other anatomical sites.

MRI further characterizes these lesions. The pericyst, representing the host’s fibrous reaction, appears hypointense on T2‐weighted sequences. Daughter cysts demonstrate hyperintensity on T2‐weighted images and hypointensity on T1‐weighted sequences. Hydatid sand typically exhibits intermediate signal intensity on T1‐weighted imaging. Postcontrast MRI reveals peripheral rim enhancement, reflecting sterile inflammatory changes in the cyst wall.

Management options for hydatid cysts include surgical excision, puncture–aspiration–injection–reaspiration (PAIR), conservative ‘watch‐and‐wait’ strategies, and pharmacological chemotherapy. Selection of the appropriate treatment modality depends on cyst size, anatomical location, stage of the disease, and presence of complications [19, 20].

#### 2.4.2. Cysticercosis

Human cysticercosis represents the larval infection caused by the cestode *Taenia solium*. The cysticercus larva can localize in various tissues, most commonly skeletal muscle, subcutaneous tissue, the central nervous system, and the eyes. Although the breast is an uncommon site for cysticercosis, several cases have been documented in the literature.

Ultrasound findings in patients with breast cysticercosis typically reveal an anechoic cyst containing an echogenic mural nodule corresponding to the scolex. Four sonographic patterns of soft tissue cysticercosis have been described: a well‐defined, round cyst with a bright echogenic protrusion from the cyst wall; a loculated fluid collection containing internal echoes and a well‐defined round cyst with an eccentric echogenic protrusion representing the scolex; an irregular‐shaped cyst with minimal fluid on one side, containing an extruded scolex; and an elliptical calcified cysticercus cyst [20].

MRI typically demonstrates cysticercosis as a lesion hyperintense on T2‐weighted images and hypointense on T1‐weighted images, with peripheral rim enhancement after contrast administration. Occasionally, the scolex appears as a T2 hypointense focus within the hyperintense cystic lesion.

FNAC is valuable for diagnosis, revealing scolices, hooklets, cyst wall fragments, and an eosinophilic inflammatory infiltrate [21–23].

#### 2.4.3. Filariasis

Filariasis is a parasitic infestation that primarily affects the lymphatic vessels and lymph nodes in humans. The most common causative organisms are *Wuchereria bancrofti*, *Brugia malayi*, and *Brugia timori*. Clinical manifestations of acute or chronic filariasis generally appear only after prolonged and repeated exposure to infected mosquito vectors. Extranodal filariasis is rare, and breast involvement represents an uncommon clinical entity [24].

In breast filariasis, lymphatic obstruction by filarial larvae results in lymphangitis, fibrosis, and disruption of lymphatic drainage. This results in a localized granulomatous inflammatory response in adjacent tissues. Gradually, progressive fibrosis replaces lymphatic vessels, leading to disruption of local lymphatic drainage. The upper outer quadrant of the breast is the most affected area, though central or periareolar nodules are also frequently reported. Lesions typically involve the subcutaneous tissue and often present as firm masses adherent to the overlying skin. Inflammatory changes, such as cutaneous oedema (peau d’orange) and axillary lymphadenopathy, may closely mimic inflammatory breast carcinoma [25].

FNAC is a definitive diagnostic tool that allows identification of microfilariae and excluding other causes in the differential diagnosis.

In the acute and subacute phases, ultrasound commonly reveals a cystic lesion with thin, echogenic, filamentous internal structures exhibiting random vigorous movements. This characteristic appearance, known as the ‘filarial dance sign’, produces a dynamic colour motion artefact on Colour Doppler imaging, first described by Amaral et al. [20, 26].

In chronic or degenerative stages, dead filarial worms undergo dystrophic calcification, which is well visualized on mammography as serpiginous or elongated calcifications that do not follow ductal anatomy. Noncalcified granulomatous lesions may also present as well‐defined nodules [27].

## 3. Noninfectious Mastitis

Noninfectious mastitis can be of inflammatory, immunological, or vascular nature.

### 3.1. Periductal Mastitis (PDM)

PDM is a chronic benign inflammatory breast disease that affects the subareolar lactiferous ducts, which are located under the nipple‐areolar complex. Histologically, it is defined by ductal ectasia, periductal fibrosis, and prominent infiltration of plasma cells, lymphocytes, and other chronic inflammatory cells. Due to the characteristic plasma cell predominance, it is also referred to as ‘plasma cell mastitis’.

The disease most frequently affects young premenopausal women, particularly those under the age of 30, or perimenopausal women, and is often associated with modifiable risk factors such as smoking, which is thought to impair ductal epithelium and contribute to squamous metaplasia.

In 1951, Zuska et al. described a specific clinical presentation now referred to as ‘Zuska’s disease’, involving recurrent subareolar abscesses and the development of mammary duct fistulae. The underlying pathophysiological process involves squamous metaplasia of the normal cuboidal epithelium lining the lactiferous ducts. This metaplastic transformation leads to keratin production, which occludes the ducts and creates a microenvironment conducive to stasis, ductal dilatation, and secondary bacterial superinfection. The obstruction of the ducts results in periductal inflammation and subsequent abscess formation, which may drain spontaneously and can develop into a periareolar cutaneous fistula [28, 29].

Multiple etiological factors have been implicated in the pathogenesis of PDM, with cigarette smoking emerging as the most consistently associated and well‐documented risk factor. Numerous clinical and pathological studies have confirmed that smoking plays a central role in the initiation and progression of the disease.

The toxic constituents of tobacco smoke, including nicotine and other carcinogenic hydrocarbons, exert direct cytotoxic effects on the ductal epithelium of the subareolar region. These agents induce epithelial injury and promote squamous metaplasia of the normal cuboidal epithelium lining the lactiferous ducts. The metaplastic squamous epithelium desquamates more readily, and the exfoliated keratinized cells may accumulate to form intraluminal plugs, leading to proximal ductal obstruction. This obstruction creates a favourable environment for bacterial colonization, particularly by anaerobic organisms, due to stagnation of secretions and localized hypoxia. In addition to direct epithelial damage, smoking‐induced alterations in hormonal signalling and vascular perfusion may contribute to the pathophysiological process, by impairing normal ductal excretion and tissue immune defence. The cumulative effect is ductal dilatation, sterile or infectious inflammation, and ultimately abscess formation. These abscesses may rupture externally and evolve into chronic draining fistulas, especially in heavy smokers, in whom both the frequency of anaerobic bacterial isolation and the risk of recurrent fistulization are notably higher [30].

The primary clinical manifestations of PDM are variable and can evolve over time, reflecting the stage and severity of the disease. Common signs and symptoms include intermittent or noncyclic breast pain, nipple discharge (which may be purulent or serous), nipple retraction or inversion, and the presence of a subareolar mass, with or without associated periareolar inflammation, abscess formation, or the development of a papillary fistula.

During the acute phase, the clinical presentation often mimics acute suppurative mastitis. Patients may experience marked erythema, swelling, warmth, and tenderness of the affected breast segment. These symptoms are frequently accompanied by abscess formation and may be associated with systemic inflammatory responses. In the subacute phase, systemic symptoms tend to subside or be absent, yet localized findings persist. Clinically, this includes the presence of palpable firm nodules, skin induration, and areas of dark red discolouration over the breast, especially in the periareolar region. In the chronic phase, PDM manifests as a persistent or slowly enlarging deep‐seated subareolar mass, often involving the areola. Chronic inflammation may lead to fibrosis, recurrent abscesses, and persistent cutaneous fistulas. [31].

The histopathology of PDM is defined as chronic inflammation of the breast, accompanied by mammary duct dilatation, plasma cell infiltration, and abscess formation. The lesion of PDM starts from the large duct in the areola area, and the duct is highly dilated microscopically. It mainly involves neutrophil infiltration in the acute phase and a large number of plasma cells and lymphocytes in the subacute phase; sometimes a large number of foam cells and multinucleated giant cells appear [32].

Mammographic findings in PDM frequently demonstrate a variety of abnormalities, most commonly including an ill‐defined mass, which may represent inflammatory tissue or abscess formation. Additionally, focal or diffuse asymmetry in breast parenchyma can be observed, reflecting underlying inflammatory changes. Retro areolar tubular or branching structures are often identified, corresponding to dilated ducts involved in the inflammatory process; these structures may sometimes exhibit dense calcifications, indicative of chronic disease and ductal epithelial damage. Such calcifications are typically linear or branching and correlate with the presence of keratinous debris or fibrosis within the ducts.

**FIGURE 5:**
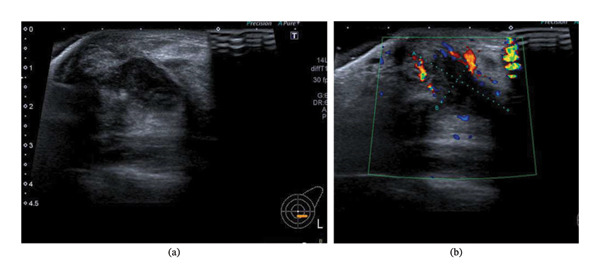
(a and b): A 28‐year‐old patient with a 1‐month history of left mastodynia, previously treated with oral antibiotics without improvement, presented with left breast enlargement, redness, and pain. Ultrasound demonstrates a hypoechoic nodule at the junction of the upper quadrants of the left breast (a), with intralesional vascularization on Colour Doppler (b). She underwent ce‐MR and an ultrasound‐guided biopsy, which revealed histological diagnosis of periductal mastitis.

**FIGURE 6:**
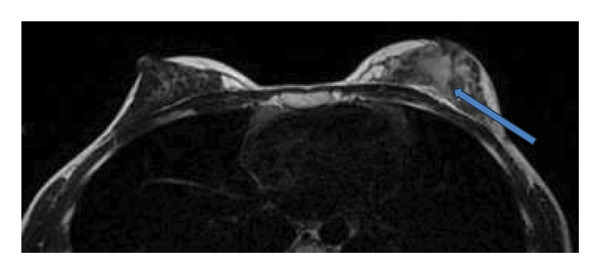
The same patient as in Figure 5. MRI T2‐weighted sequence demonstrates diffuse edema of the left breast parenchyma, with some internal fluid component extending to the inverted nipple. On postcontrast imaging, there is a diffuse increased enhancement of the entire left breast parenchyma in the ce‐T1 sequence, with an abscess‐like formation in continuity with the lactiferous duct and associated with nipple inversion. The patient recovered after intravenous antibiotic therapy with clindamycin.

**FIGURE 7:**
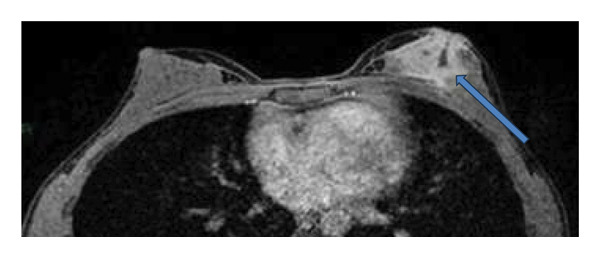
Same patient as in Figure 5‐6. On post‐contrast imaging, there is a diffuse increased enhancement of the entire left breast parenchyma in the ce‐T1 sequence, with an abscess‐like formation in continuity with the lactiferous duct and associated with nipple inversion. The patient recovered after intravenous antibiotic therapy with clindamycin.

On ultrasound imaging, the findings are variable but often include complex cystic lesions that contain both fluid and solid components, as well as nonspecific heterogeneously hypoechoic masses with ill‐defined borders. A hallmark feature is the presence of dilated subareolar ducts, which frequently contain intraluminal echogenic debris consistent with inspissated secretions, keratin, or inflammatory exudate. The duct walls usually exhibit increased echogenicity and thickening, reflecting periductal inflammation and fibrosis. Moreover, Colour Doppler ultrasound typically reveals increased vascular flow in the periductal breast tissue, indicative of active inflammation and hyperaemia. In chronic cases, long‐standing duct ectasia often leads to the formation of calcified plugs within the duct lumens. These calcifications appear as multiple discrete hyperechoic foci, often linear or branching in morphology, and are associated with posterior acoustic shadowing on ultrasound [8, 11, 33] (Figures 5, 6, and 7).

### 3.2. Idiopathic Granulomatous Mastitis (IGM)

IGM is a form of benign chronic breast inflammation with unknown pathogenesis, characterized by noncaseating granuloma and microabscess formation, limited to lobules of the mammary gland [34].

There are three hypotheses responsible for the pathogenesis of most IGM cases: autoimmunity, infection, and hormone disorders.

The frequent presence of lower extremity erythema in patients with granulomatous lobular mastitis (GLM) supports the idea that this condition may have an autoimmune origin. GLM has also been reported in association with systemic symptoms like erythema nodosum and arthritis, further suggesting an immune‐mediated process.

Corynebacterium species, particularly *C. kroppenstedtii*, are increasingly recognized as possible pathogens in IGM, although their precise role is debated, as they are part of the normal skin flora [6].

Research suggests that high levels of prolactin may play a direct role in the development of GLM. Elevated prolactin levels may enhance ductal secretions, potentially causing damage to the ductal epithelium and allowing milk‐like secretions to leak into the surrounding interlobular stroma. This extravasation can trigger a granulomatous inflammatory response localized around the lobules, contributing to the development of granulomatous mastitis.

It is also known that hormones like prolactin may disrupt the immune balance within breast tissue, stimulating epithelial cells to secreting pro‐inflammatory factors, and leading to an inflammatory response of interlobular stroma and eventual abscess formation [35].

Histologically, GLM is a lobular inflammatory condition defined by noncaseating granulomas that include epithelioid histiocytes, multinucleated giant cells, lymphocytes, plasma cells, and neutrophils. Fat necrosis and sterile microabscesses are common, with granulomas centred on the lobules. The condition can also involve loss of acinar structures in affected ducts and lobules, with inflammation potentially extending to adjacent lobules in more severe cases [6].

The most common clinical presentation of IGM is a unilateral, tender palpable mass within the breast. It can also present as multiple simultaneous areas of peripheral, rarely central, masses or inflammation. The lesions may occur in any quadrant of the breast, with the subareolar region being the least affected. Symptoms can persist from 6 months to 2 years, but the disease tends to be self‐limiting.

The mammographic findings of IGM are nonspecific. The most frequently encountered mammographic appearance is focal asymmetry. Alternatively, it can present as one or more irregularly shaped masses. The area of IGM can also be associated with heterogeneous calcifications [36].

The sonographic features of IGM typically include lobulated, heterogeneous, hypoechoic masses with indistinct, irregular, or angular margins. Common findings also include indistinct hyperechoic rims and increased internal vascularity. Tubular extensions are frequently observed and may correspond to involvement of the segmental ductal‐lobular system and surrounding perilobular inflammation. Additional ultrasound findings can include skin thickening, oedema, obliteration of subcutaneous fat, and the presence of sinus tracts extending to the skin or between lesions. Most masses are located in the peripheral breast [8].

Breast MRI may sometimes be helpful to document the extent and progression of disease. Heterogeneously enhancing or rim‐enhancing masses are the most common findings. MRI can also characterize nipple retraction, sinus tracts, and parenchymal distortion, but it cannot reliably distinguish breast cancer from granulomatous mastitis—so that histological diagnosis with percutaneous needle biopsy remains mandatory [37].

While pathologies in the subareolar area suggest PDM, lesions away from the nipple are more likely IGM. Acute cases that improve with treatment are usually infectious, instead persistent ones are more likely to be chronic. Biopsy is required in prolonged or recurrent cases that do not respond to therapy. Moreover, infectious, noninfectious, and tumour‐related inflammations often overlap, and sharp boundaries do not exist. For example, in IGM or inflammatory breast cancer (IBC), the skin layer is fragile, and microbial agents may be added.

**FIGURE 8:**
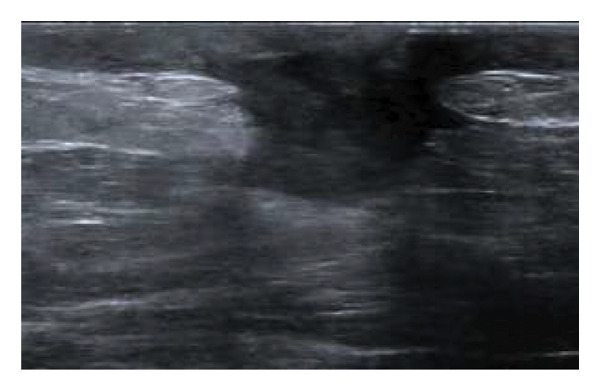
A 44‐year‐old patient presented with left breast mastodynia. Ultrasound demonstrates a hypoechoic nodular formation with irregular margins in the retro areolar region, inseparable from the areola–nipple complex, which appears markedly thickened (8). The Colour Doppler examination shows increased peripheral vascular signals (9).

**FIGURE 9:**
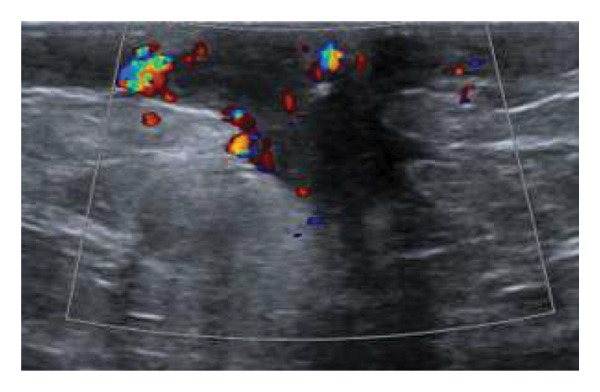
Same patient as in Figure 8. The color Doppler examination shows increased peripheral vascular signals (9).

**FIGURE 10:**
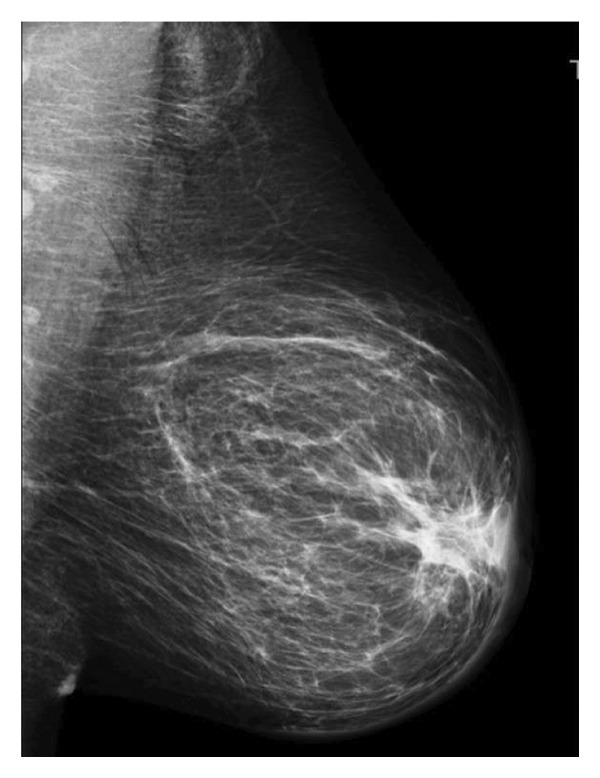
The same patient as in Figures 8 and 9. The oblique medio‐lateral projection of the left breast shows an extensive area with poorly defined margins in the retro areolar area, with faint calcifications in the context inseparable from the areola‐nipple complex, which appears retracted; these findings are associated with diffuse thickening of the skin layer.

**FIGURE 11:**
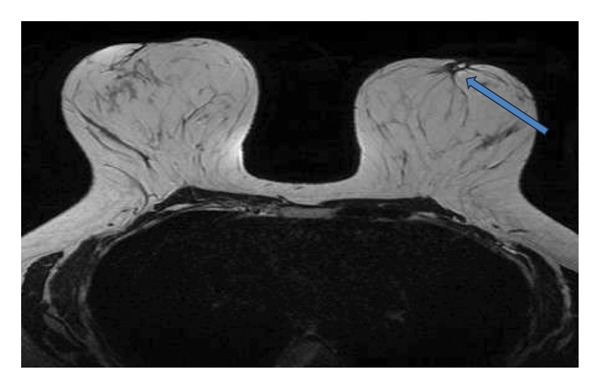
The patient (the same as in Figures 8, 9, 10) underwent ce‐MRI examination. The T2‐weighted sequence shows a small left retro areolar fluid collection, associated with distortion of the adjacent breast parenchyma (arrow in 11).

**FIGURE 12:**
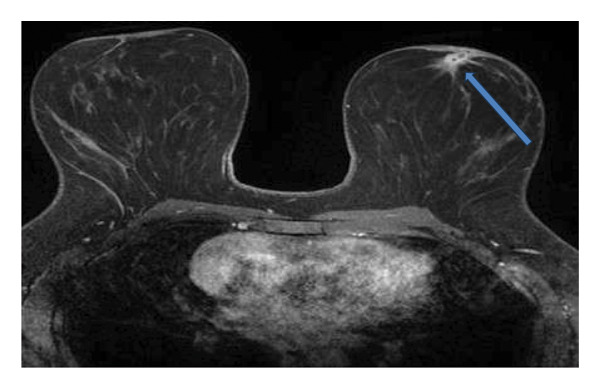
Same patient as in Figure 11. The post‐contrast T1‐sequence demonstrates in the same region a distorted area of altered enhancement with “mass‐like” pattern, with hypointense content on T1 due to its fluid nature and with thickened margins and enhancement, resembling an abscess (arrow in 12). Ultrasound‐guided biopsy revealed histological features consistent with idiopathic granulomatous mastitis (IGM).

The treatment of IGM remains controversial. The management is generally tailored to each patient’s clinical manifestations. Treatment options include conservative measures such as close regular surveillance and medication therapy with corticosteroids or methotrexate, and the more aggressive approach of wide local surgical excision [38] (Figures 8, 9, 10, 11, and 12).

### 3.3. Diabetic Mastopathy

Diabetic mastopathy, also known in the literature as ‘lymphocytic mastopathy’, ‘fibrocystic mastopathy’, or ‘fibrocystic breast degeneration’, is a distinct clinicopathologic entity characterized by dense fibrous proliferation and lymphocytic infiltration of the breast stroma. This rare condition predominantly affects young to middle‐aged women with a prolonged history of type 1 diabetes mellitus.

The pathophysiological mechanisms underlying diabetic mastopathy remain incompletely understood, though several immunologic and metabolic hypotheses have been proposed. One prevailing theory suggests an autoimmune aetiology, as histopathological examination frequently reveals periductal and perivascular aggregates of B‐lymphocytes expressing HLA‐DR class II antigens within affected breast tissue, indicating an antigen‐driven immune response [39].

Tomaszewski et al. hypothesized that chronic hyperglycaemia induces nonenzymatic glycosylation of extracellular matrix proteins, leading to their accumulation and structural modification. These glycosylated matrix components may act as neoantigens, triggering a localized autoimmune reaction characterized by B‐cell proliferation and the production of specific autoantibodies directed against altered matrix molecules [40].

In addition, an alternative mechanism proposed by Seidman et al. implicates the body’s inflammatory response to exogenous insulin therapy as a potential trigger. According to this hypothesis, repeated insulin administration may incite localized immune activation, contributing to the fibroinflammatory changes observed in diabetic mastopathy [41].

Diabetic mastopathy typically presents clinically as a painless, palpable breast mass. In advanced stages, bilateral breast involvement may be observed, reflecting the progressive nature of the disease.

Mammographically, diabetic mastopathy is often characterized by ill‐defined, dense solid lesions, focal asymmetric densities, or architectural distortions. These imaging features are frequently indistinguishable from malignant breast neoplasms. On ultrasound examination, the condition manifests as irregular, hypoechoic nodular masses, exhibiting marked posterior acoustic shadowing, attributable to the dense fibrotic stroma. The lesion size may vary considerably, ranging from approximately 5 to 60 mm in diameter. Less commonly, especially in the early stages of the disease when fibrotic changes are minimal, diabetic mastopathy may appear as well‐circumscribed or mildly hypoechoic masses lacking prominent acoustic shadowing [26, 39].

In recent years, contrast‐enhanced MRI has been proposed as a supplementary imaging modality in the evaluation of diabetic mastopathy. The pathological appearance of diabetic mastopathy on MRI is highly variable, reflecting the heterogeneous histological nature of the disease. Reported enhancement patterns include slow, progressive uptake of contrast as well as more rapid and intense enhancement, both of which may resemble imaging features typically observed in malignant breast tumours. This overlap poses a significant diagnostic challenge and limits the specificity of MRI in distinguishing benign from malignant lesions in diabetic patients [42].

**FIGURE 13:**
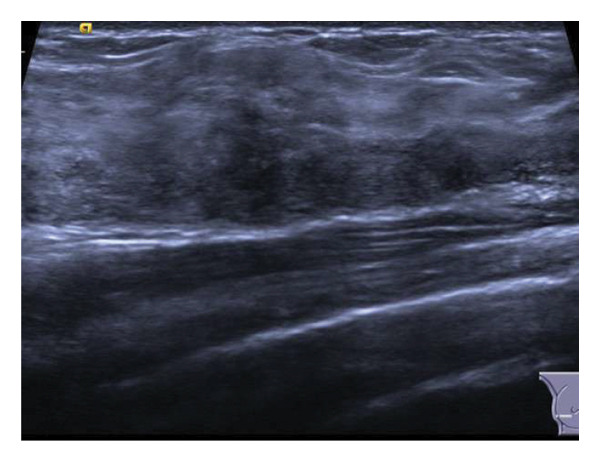
A 35‐year‐old patient with diagnosis of type 1 diabetes mellitus presented for further evaluation of a hypoechoic area with posterior acoustic attenuation at breast ultrasound, corresponding to a clinically evident swelling in the lower outer quadrant of the right breast.

**FIGURE 14:**
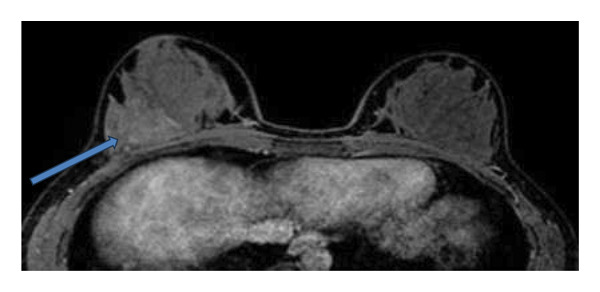
The same patient as in Figure 13. Post‐contrast MRI sequences demonstrate a non‐mass‐like regional area of weak and delayed enhancement in the lower outer quadrant of the right breast (arrow).

**FIGURE 15:**
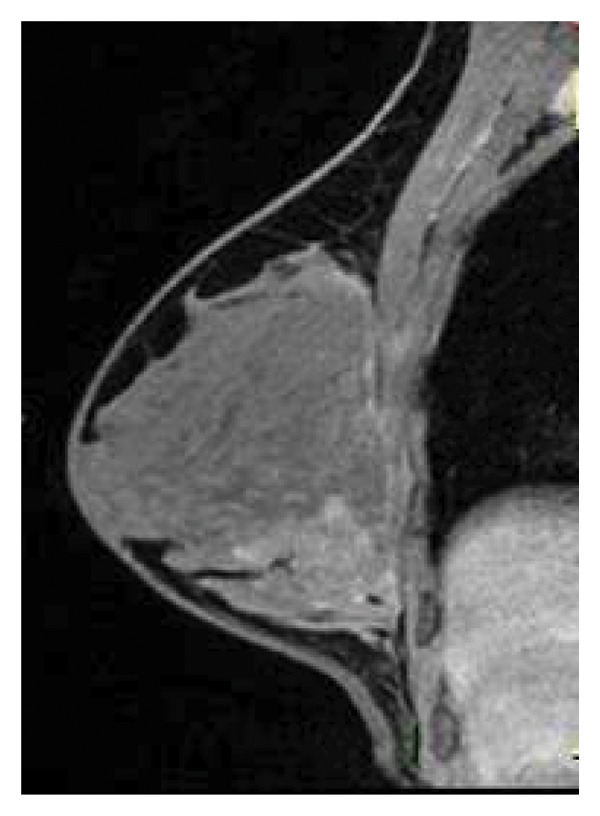
Same patient as in Figures 13 and 14. The sagittal post‐contrast MRI sequence demonstrates a non‐mass‐like regional area of mainly delayed enhancement in the lower outer quadrant of the right breast. The ultrasound‐guided biopsy revealed histological features consistent with diabetic mastopathy.

Given the considerable overlap in clinical and imaging features between diabetic mastopathy and invasive breast carcinoma, histopathological confirmation remains indispensable for accurate diagnosis. CNB or excisional biopsy can provide definitive tissue diagnosis; however, due to the high rate of recurrence following surgical excision, reported in up to 60% of cases, complete surgical biopsy is generally discouraged [39] (Figures 13, 14, and 15).

### 3.4. Mondor’s Disease

Mondor’s disease is a superficial thrombophlebitis involving the subcutaneous veins of the anterolateral thoracoabdominal wall, most frequently affecting women between the third and fifth decades of life. When localized to the breast, it is referred to as Mondor’s disease of the breast (MDB).

Approximately 50%–60% of cases are idiopathic, whereas 40%–50% are associated with predisposing factors. The most reported secondary causes are trauma and iatrogenic interventions, such as surgery, biopsy, or tight clothing. Importantly, Mondor’s disease can also be associated with underlying breast malignancy, and its presence does not exclude the possibility of breast cancer.

Clinically, patients typically present with localized pain or tenderness along the lateral aspect of the breast or chest wall, accompanied by a palpable, cord‐like subcutaneous induration. The diagnosis is often clinical, supported by characteristic findings on imaging. Mammography is frequently normal but may reveal a superficial tubular density corresponding to the affected vein. Ultrasonography shows a superficial, noncompressible, hypoechoic tubular structure without internal flow on Colour Doppler imaging, consistent with a thrombosed vein.

**FIGURE 16:**
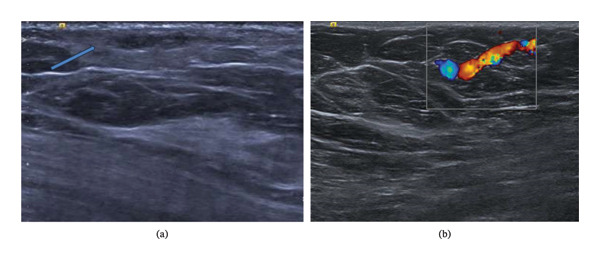
(a and b): A 70‐year‐old patient with a history of left breast carcinoma reported mastodynia at the junction of the upper quadrants of the right breast. The ultrasound shows an anechoic area with a cord‐like morphology in the superficial area (arrow in a) corresponding to a palpable nodule without internal flow on Colour Doppler imaging, consistent with a thrombosed vein. In b, the same vein after resolution of the thrombosis: there is a colour signal in the vessel on the Colour Doppler module.

Mondor’s disease is generally self‐limiting, with spontaneous resolution occurring within 4 to 8 weeks. Management is conservative and includes nonsteroidal anti‐inflammatory drugs and analgesics for symptomatic relief. Follow‐up imaging may be warranted in persistent or atypical cases to rule out underlying pathology [9, 43, 44] (Figure 16).

## 4. Inflammation Related to Breast Malignancy

### 4.1. IBC

Inflammatory cancer is an aggressive form of invasive cancer characterized by diffuse infiltration of breast tissue and the sudden onset of symptoms, that generally occurs in older women (average age: 65 years) [45].

The 8th edition of the American Joint Committee on Cancer (AJCC) staging manual continues to define IBC based upon the presence of diffuse erythema and oedema involving a third or more of the breast after confirmation of an invasive breast cancer. The diagnosis of IBC is categorized as clinical stage T4d [46].

Typically, IBC is diagnosed within 6 months of the onset of clinical symptoms. Patients present with diffuse breast oedema visible by clinical examination with ‘orange peel’ skin or erythema. The entire breast is usually swollen, and no real palpable masses are detected. The typical clinical presentation of skin inflammation is a consequence of tumour cell emboli within the lymphatic vessels that are mainly subcutaneous in the breast [47].

The defining pathological feature of IBC is the presence of tumour cell emboli within the dermal lymphatic vessels of the papillary and reticular dermis. It is thought that the dermal lymphatic emboli obstruct lymphatic drainage in the skin, leading to the characteristic clinical signs of IBC, including erythema, oedema, and peau d’orange. Beyond their role in local inflammation, dermal lymphatic emboli may also contribute to regional tumour spread. Some experimental models have raised the possibility that these emboli could serve as an additional source of distant metastasis, independent of the primary tumour, reflecting the highly aggressive nature of IBC [48].

Mammography is the initial imaging modality of choice in suspected IBC and should always be performed bilaterally to compare both breasts and assess for additional pathology. Typical findings include skin thickening, often starting in the areolar region, stromal coarsening, trabecular thickening, and diffuse parenchymal density or asymmetry. Focal masses, architectural distortion, or microcalcifications are less common.

Ultrasound is equally important in early assessment. High‐frequency probes can detect most lesions and guide CNB for histological confirmation. Common sonographic features include diffuse skin thickening, parenchymal hyperechogenicity due to oedema, distorted Cooper’s ligaments, and irregular hypoechoic masses with posterior shadowing and increased vascularity. It also assesses nodal involvement, including axillary, parasternal, and supraclavicular nodes.

**FIGURE 17:**
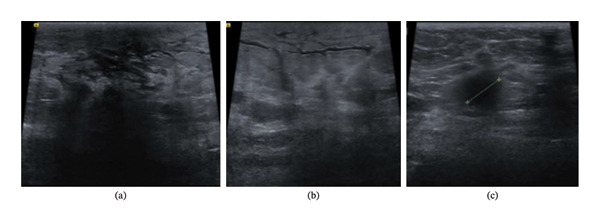
(a, b and c): A 43‐year‐old patient with diagnosis of left breast carcinoma presented with left mastodynia for 2 months. The ultrasound of the affected left breast shows diffuse subcutaneous oedema with thickening of the skin layer (a and b). In c, a hypoechoic, globular, ipsilateral axillary adenopathy is shown, without recognizability of the adipose hilum.

**FIGURE 18:**
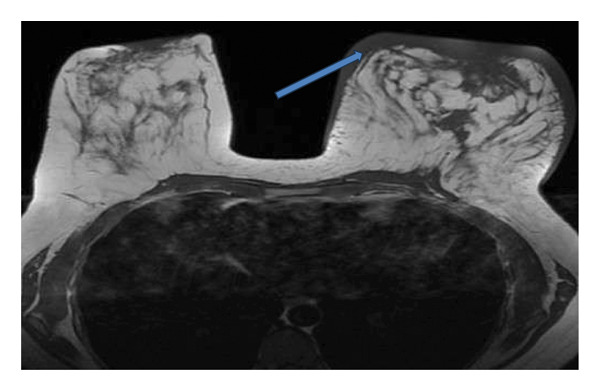
The same patient as in Figure 17. The MRI T2‐weighted sequence shows breast asymmetry, with the left breast larger in size and associated with diffuse thickening of the skin (18).

**FIGURE 19:**
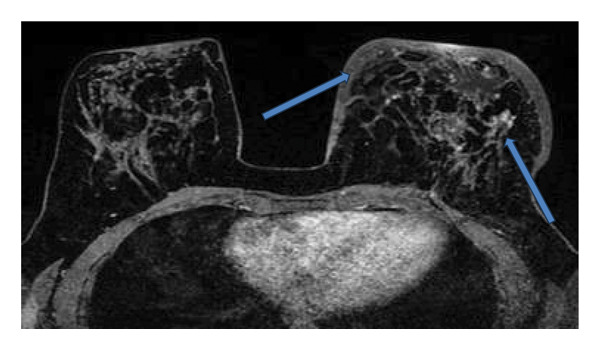
Same patient as in Figure 17‐18. The post‐contrast T1 sequence demonstrates a multifocal area of altered enhancement of the left breast with extension to the areola‐nipple complex, which appears infiltrated, thickened and retracted (arrows in 19).

MRI is the most sensitive modality for detecting and staging IBC. It provides detailed information on lesion extent, skin and chest wall involvement, contralateral disease, and lymph node status. Characteristic MRI findings include diffuse skin thickening with enhancement, parenchymal and chest wall oedema, and either a heterogeneous mass or nonmass enhancement, often reflecting multifocal or confluent disease rather than a single dominant lesion [49] (Figures 17, 18, and 19).

## 5. Conclusions

The differential diagnosis of mastitis requires a strong clinical framework, as well as knowledge of the radiological presentation of the various clinicopathological entities. Mastitis is classified into infectious and noninfectious forms, including inflammatory conditions associated with or overlapping with cancer. IBC is a rare but aggressive entity that can present with clinical signs similar to mastitis. Although IBC can be difficult to recognize, the failure to respond to antibiotic therapy, persistent clinical signs, and certain imaging patterns (especially MRI) should always raise suspicion of cancer and prompt further histological investigations. Very often, inflammatory breast diseases regress spontaneously with proper treatment, so a conservative approach is preferable to surgery (as surgery is not free of complications). Surgery should be reserved for cases where mammogram and ultrasound results are questionable, inflammation symptoms persist despite antibiotic and corticosteroid therapy, and in the presence of palpable masses. Furthermore, an interventional approach with US‐guided biopsy is necessary before surgery, in order to obtain a histological sample that supports the diagnosis. In conclusion, proper integration of clinical, imaging, and follow‐up care is essential to avoid unpleasant diagnostic delays. The radiologist plays a key role in recognizing signs suggestive of benign or malignant disease, guiding clinical decisions, and ensuring appropriate and timely patient management.

## Author Contributions

Conceptualization: Cristiana Boldrini; data curation: Cristiana Boldrini, Micol Bottalico and Francesca L. Lia; formal analysis: Cristiana Boldrini; investigation, methodology and project administration: Cristiana Boldrini; resources: Micol Bottalico; supervision and validation: Cristiana Boldrini; writing–original draft: Cristiana Boldrini, Micol Bottalico and Francesca L. Lia; writing–review and editing: Cristiana Boldrini.

## Funding

The authors declare that no funds, grants, or other support were received during the preparation of this manuscript. Open access publishing facilitated by Universita Cattolica del Sacro Cuore, as part of the Wiley ‐ CRUI‐CARE agreement.

## Conflicts of Interest

The authors declare no conflicts of interest.

## Data Availability

The data that support the findings of this study are available in PubMed at https://pubmed.ncbi.nlm.nih.gov/. These data were derived from the following resources available in the public domain: https://pubmed.ncbi.nlm.nih.gov/, https://pubmed.ncbi.nlm.nih.gov/
